# Advances in Biointegrated Wearable and Implantable Optoelectronic Devices for Cardiac Healthcare

**DOI:** 10.34133/cbsystems.0172

**Published:** 2024-10-18

**Authors:** Cheng Li, Yangshuang Bian, Zhiyuan Zhao, Yunqi Liu, Yunlong Guo

**Affiliations:** ^1^ Beijing National Laboratory for Molecular Sciences, Key Laboratory of Organic Solids, Institute of Chemistry, Chinese Academy of Sciences, Beijing 100190, China.; ^2^ School of Chemical Sciences, University of Chinese Academy of Sciences, Beijing 100049, China.

## Abstract

With the prevalence of cardiovascular disease, it is imperative that medical monitoring and treatment become more instantaneous and comfortable for patients. Recently, wearable and implantable optoelectronic devices can be seamlessly integrated into human body to enable physiological monitoring and treatment in an imperceptible and spatiotemporally unconstrained manner, opening countless possibilities for the intelligent healthcare paradigm. To achieve biointegrated cardiac healthcare, researchers have focused on novel strategies for the construction of flexible/stretchable optoelectronic devices and systems. Here, we overview the progress of biointegrated flexible and stretchable optoelectronics for wearable and implantable cardiac healthcare devices. Firstly, the device design is addressed, including the mechanical design, interface adhesion, and encapsulation strategies. Next, the practical applications of optoelectronic devices for cardiac physiological monitoring, cardiac optogenetics, and nongenetic stimulation are presented. Finally, an outlook on biointegrated flexible and stretchable optoelectronic devices and systems for intelligent cardiac healthcare is discussed.

## Introduction

In recent years, biointegrated optoelectronic devices have received considerable attention as the key component of wearable and implantable systems. High-performance optoelectronic devices, such as light-emitting diodes (LEDs) and photodetectors (PDs), have been widely used in communications, medical healthcare, etc. [1–3]. However, the incompatibility between conventional rigid devices and biological systems poses huge challenges for reliable biointegration. Generally, the elastic modulus of biological tissues (e.g., brain, heart, and skin) is about 1 to 10 kPa, whereas the elastic modulus of rigid optoelectronic devices is typically 10 to 100 GPa [4]. Large mechanical mismatch may cause severe discomfort and side effects such as inflammation [5]. To address this problem, it is essential to improve the flexibility or even stretchability of optoelectronic systems. Structural and strain engineering are considered to be effective strategies for conferring flexibility and stretchability to rigid electronics [6–8]. Furthermore, the design of intrinsically flexible/stretchable materials and devices has also attracted much attention due to their high durability and superior optoelectronic properties even at large strain or high-integration levels [9–11]. It is anticipated that the flexible and stretchable optoelectronic systems developed using these approaches will facilitate seamless integration with highly dynamic organs without imposing mechanical constraints on the natural motion of soft tissues [12].

The incidence of cardiovascular diseases (CVDs) has been increasing in recent years, and CVDs are responsible for 31% of global deaths [13,14]. Despite the high death rate, 90% of CVDs can be prevented through early detection. Therefore, continuous and real-time cardiac physiological monitoring is essential to reduce the morbidity and mortality of CVDs. However, current clinical instruments are bulky and intermittent, making it difficult to perform real-time monitoring without spatiotemporal restrictions. Flexible and stretchable optoelectronic devices have demonstrated huge advantages in the prevention and diagnosis of CVDs [15–17]. For example, these devices are relatively small, thin and lightweight, allowing for attaching on human skin or implanting in the body imperceptibly for continuous cardiac physiological monitoring without spatial and temporal constraints [16]. They also allow conformal contact with tissues or organs, thus optimizing the signal-to-noise ratio and minimizing motion artifacts for continuous cardiac physiological monitoring [18,19]. Therefore, the development of wearable and implantable optoelectronic devices can provide long-term personalized prevention, diagnosis, and treatment strategies, thus enabling the smart cardiac healthcare paradigm.

In addition to cardiac monitoring, flexible and stretchable optoelectronic devices are also employed in the therapy of cardiac diseases such as arrhythmias and heart failure [20–23]. Currently, electrical pacing and defibrillation are commonly used to restore normal cardiac rhythm. For example, implantable bioelectronic devices such as multielectrode arrays have been used as cardiac pacemakers to treat ventricular arrhythmias [24–26]. To achieve higher cell specificity and spatiotemporal resolution, optogenetics have been developed for rhythm control with ultralowenergy consumption, precise autonomic nervous modulation, and painless defibrillation [27–29]. However, due to the limited distance of light penetration and the diffuse nature of the LED light, high-resolution optoelectronic systems are needed to be implanted to shorten the light transmission distance and achieve precise light delivery. Apart from cardiac optogenetics, nongenetic cardiac stimulation systems based on the photoelectric conversion effect are being developed in parallel to enable precise light-controlled electrical stimulation without complicated genetic engineering [30–32].

**Fig. 1.**
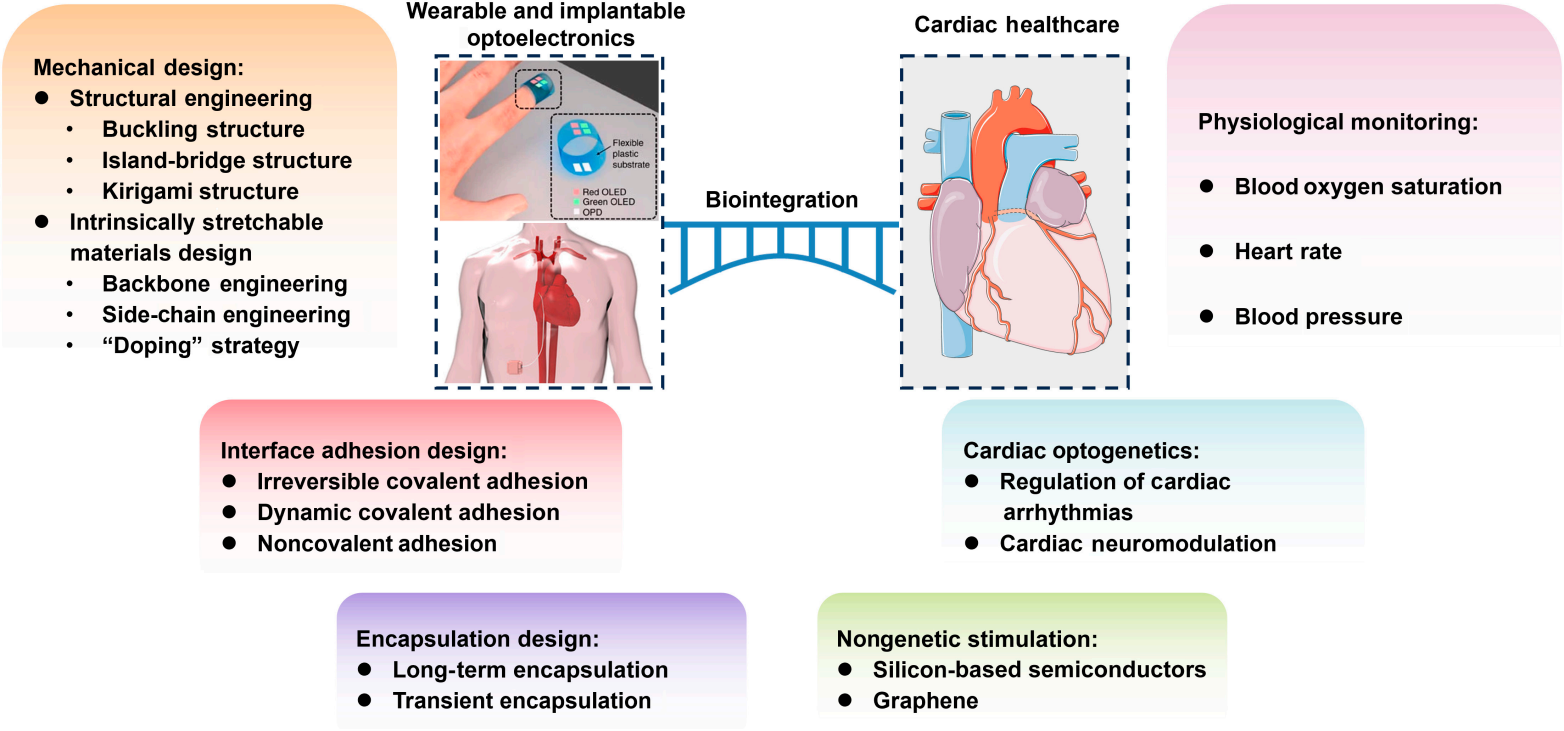
Biointegrated wearable and implantable optoelectronic devices for cardiac healthcare. Mechanical, interface adhesion, and encapsulation design strategies for wearable and implantable optoelectronic devices (left). Cardiac healthcare applications of wearable and implantable optoelectronic systems for cardiac physiological monitoring, cardiac optogenetics, and nongenetic stimulation (right). The figures in “Wearable and implantable optoelectronics”. Reproduced with permission from [91]. Copyright 2014, Springer Nature Limited (top). Reproduced under the terms of the Creative Commons CC-BY-NC license from [95]. Copyright 2021 The American Association for the Advancement of Science (bottom).

In this review, we focus on the advances in biointegrated flexible and stretchable optoelectronics for wearable and implantable cardiac healthcare systems (Fig. 1). Firstly, we highlight stretchable design strategies (structural engineering and intrinsically stretchable material design) to achieve conformal contact with highly curved and dynamic tissues and organs. Interface adhesion design strategies between devices and biological tissues are also summarized for enabling strong adhesion and functional exchange at the interfaces. Additionally, long-term and transient encapsulation strategies are also discussed. Next, we review the advances in wearable and implantable optoelectronic systems for monitoring blood oxygen saturation (SpO_2_), heart rate (HR), and blood pressure (BP). We then move on to optoelectronic systems for optogenetics-based HR regulation and neuromodulation. Nongenetic optoelectronic stimulation of hearts is also briefly discussed. Finally, an outlook on the challenges of biointegrated flexible and stretchable optoelectronic devices for cardiac healthcare is provided.

## Device Designs for Biointegrated Optoelectronics

Distinguished from conventional electronic/optoelectronic devices, biointegrated devices require additional design strategies to fulfill their functions in biointegrated systems. Firstly, achieving mechanical matching between devices and human organs can circumvent the adverse immune responses and degradation of device performance [33,34]. Therefore, it is necessary to design optoelectronic devices with stretchability to achieve mechanical coupling and conformal contact with biological systems, especially for highly dynamic organs. Secondly, the physical interface between the electronic system and the biological system must provide reliable, uniform, and time-independent adhesion to both the biological tissue and the electronic device over the entire interfacial region [35–37]. Thirdly, it is important to rationally design the encapsulation layer that can effectively block the penetration of ion-rich biofluids and protect the optoelectronic devices from damage. In this section, we focus on the design of biointegrated optoelectronic devices in terms of stretchability, interface adhesion, and encapsulation strategies.

### Mechanical design for stretchability

Flexibility and even stretchability are indispensable elements for implantable optoelectronic devices. Firstly, the vast majority of biological tissues and organs are low-modulus; thus, only optoelectronic devices with moduli similar to those of biological organs can form low-stress and tight bioelectronic interfaces. Secondly, most biological tissues and organs have surfaces with nonzero Gaussian curvature, whereas optoelectronic devices are typically fabricated as planar structures. Therefore, the optoelectronic devices require high stretchability for conformal attachment to the organ surface. Thirdly, highly dynamic organs, such as the heart, are subject to constant and irregular deformation and motion. In order to maintain their functionality under repeated strains in all directions, optoelectronic devices require to be endowed with high stretchable properties.

At present, there are 2 main strategies to endow devices with stretchability: (a) physical or structural stretchability and (b) intrinsically stretchability. The first strategy is to employ structural or strain engineering on rigid materials. The commonly employed design strategies include the buckling structure [6,38,39], island-bridge structure [7,40,41], and kirigami structure [8,42,43].

#### Structural engineering

**Fig. 2.**
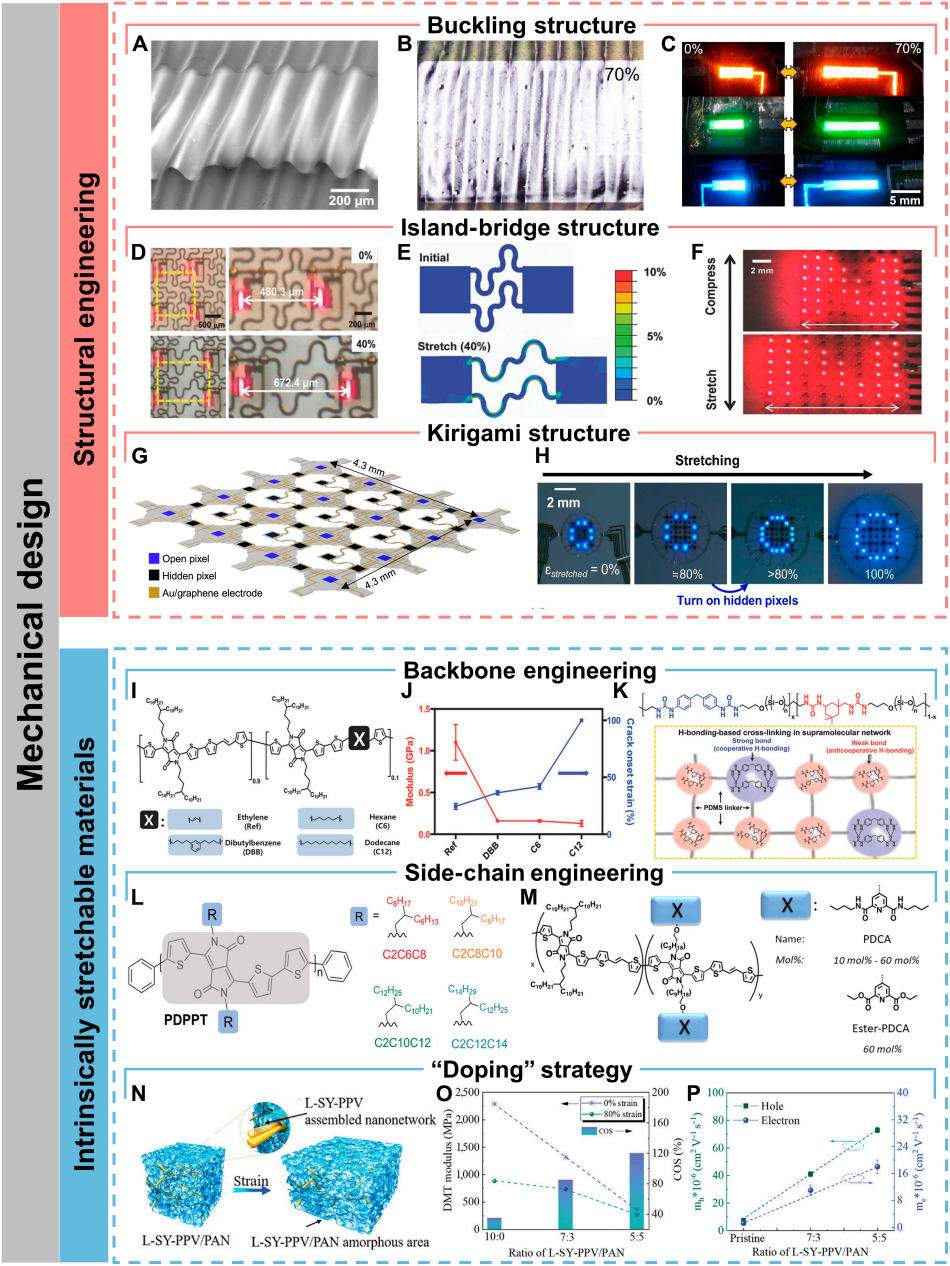
Mechanical design for stretchability. (A) Perspective scanning electron micrograph of a buckling-structured LED. (B) Optical image of the buckling-structured LED under 70% tensile strain along the horizontal direction. (C) Photographs of the stretchable red, green, and blue buckling-structured LEDs at strains of 0% and 70%, respectively. Reproduced with permission from [39]. Copyright 2017 American Chemical Society. (D) Stretchable μ-LEDs adopting the island-bridge approach. (E) Strain distribution simulated by finite element method on the stretchable μ-LEDs based on island-bridge structure. (F) Photography of the stretchable μ-LEDs under uniaxial strain (top 0%, bottom 40%). Reproduced with permission from [48]. Copyright 2017 WILEY-VCH Verlag GmbH & Co. KGaA, Weinheim. (G) Schematic illustration of morphable displays transforming from 2D precursors to 3D structures under strain. (H) Optical images show the morphable display with the real-time automatic feedback system exhibiting character “O” when stretching. Reproduced with permission from [42]. Copyright 2022 Elsevier Ltd. (I) Chemical structures of DPP-based semiconducting polymers containing 10 mol% of conjugation breakers. (J) Elastic modulus and crack onset strain of the polymer semiconductors. Reproduced with permission from [56]. Copyright 2018 WILEY-VCH Verlag GmbH & Co. KGaA, Weinheim. (K) Chemical structure of PDMS–MPU_x_–IU_1−x_ (top) and possible hydrogen bonding combinations for strong bond and weak bond, respectively (bottom). Reproduced with permission from [65]. Copyright 2018 WILEY-VCH Verlag GmbH & Co. KGaA, Weinheim. (L) Schematics of PDPPT polymers with different side-chain structures. Reproduced with permission from [59]. Copyright 2021 Wiley-VCH GmbH. (M) Schematics of the chemical structure of the DPP-based polymers and their respective side-chain H-bonding groups 2,6-pyridinedicarboxamide (PDCA) units. Reproduced with permission from [60]. Copyright 2019 American Chemical Society. (N) 3D schematic of the morphology comprising embedded 3D penetrating nanonetworks of L-SY-PPV/PAN. (O) Derjauin−Muller−Toporov (DMT) modulus and the crack onset strain (COS) results for the L-SY-PPV/PAN mixed film with different PAN contents. (P) Hole and electron mobilities in the L-SY-PPV/PAN mixed film with different PAN contents. Reproduced with permission from [63]. Copyright 2022 Wiley-VCH GmbH.

Buckling structure: Stretchable optoelectronic devices with buckling structures typically are created by depositing rigid materials onto an ultrathin film substrate and subsequently transferring both together onto a prestretched polymer elastomer substrate [1,44]. When the prestrain is released, the rigid but bendable ultrathin film is subjected to compressive stresses due to the retraction of the elastomer substrate, resulting in periodic wrinkle structure (Fig. 2A). During the stretching process, these wrinkles are gradually flattened (Fig. 2B), resulting in a degree of stretchability up to the level of the prestrain [6]. Yao et al. [38] achieved the buckling structure by laminating the device on a prestretched acrylic tape-silicon rubber sheet and subsequently transferred the prepared stretchable organic LED (OLED) onto a prestretched (300% of the initial length) rubber substrate. The device could withstand up to 100% tensile strain, and even mechanical deformation of 90% tensile strain did not lead to dramatic degradation of electroluminescence performance, with current efficiency and brightness remaining above 85%. Kim et al. [39] fabricated a buckling-structured stretchable photosensor by transferring a colloidal quantum dot-based photodiode and a LED onto a prestrained elastomeric substrate for continuous recording of photoplethysmography (PPG) signal pulses. The stretchable photosensor could be stretched to 70% strain without degradation of device performance and could be folded to a bend radius of curvature of 35 μm (Fig. 2C).

Island-bridge structure: Originally proposed by Rogers et al. [40], the island-bridge structure has been most widely used for stretchable optoelectronic devices, which have already achieved high optoelectronic performance at large strains [45,46]. Typically, rigid and functional optoelectronic devices are integrated on stretchable substrates and linked by interconnects with stretchable structures design (e.g., serpentine and wavy structures) to form island-bridge structure [47,48] (Fig. 2D). During stretching, the structurally designed interconnects are able to efficiently dissipate the strain applied to the entire system, while the rigid islands only suffer less than 1% of the strain [49] (Fig. 2E). However, there are drastic strain variations at the stretchable–rigid interfaces (elastomeric substrate and rigid islands, stretchable interconnects and rigid islands), which may induce separation of the optoelectronic devices from the elastomeric substrate or interconnects [50]. Yang et al. [41] developed and optimized a “Ferris wheel-shaped island”, which could effectively suppress crack propagation at the interface between the island and the stretchable substrate through an interlocking structure. With the “Ferris wheel-shaped island” design, no substantial damage was observed at the interface even when the tensile rate reached ~175%. The “Ferris wheel island” not only effectively inhibited crack propagation and improved the stretchable property but also enhanced the fatigue resistance of the devices. No interface damage was observed after 1,000 cycles at 120% strain. Stretchable LED arrays were fabricated by integrating rigid “islands” and serpentine Ag electrodes into stretchable electronic devices. The electrodes had good interfacial stability, maintaining a low resistance of 27 Ω at a high strain rate of 220%, and the arrays performed well in all 3-dimensional (3D) deformation modes.

Although island-bridge structure can maintain the performance of rigid optoelectronic devices well, in most cases, the interconnections occupy more than 50% of the surface area, resulting in a low filling factor and device density [51] (Fig. 2F). Therefore, island-bridge structure may not be the optimal choice for applications that require high resolution and maximized device encapsulation.

Kirigami structure: In comparison to island-bridge structure, kirigami structure, inspired by the art of paper cutting, have a higher filling factor and enhanced deformation tolerance [5]. During the initial elastic phase, the mechanical stresses are concentrated at the connection nodes. As stresses gradually increase, the kirigami structure can minimize the strains on the optoelectronic device through bending and torsional deformations throughout the system. Kirigami structures can give optoelectronic devices high stretchability far beyond the deformation limits of the material or the device itself, as well as giving planar devices a variety of 3D deformability, such as multidirectional bending, curling, and twisting capabilities [52]. Lee et al. [42] constructed a stretchable display based on the kirigami 3D structure design that could maintain pixel density and image quality at 100% high strain (Fig. 2H). They placed hidden micro-LED (μ-LED) pixels at specific locations, hidden in the absence of strain and exposed in the biaxial stretching state to supplement the pixel density (Fig. 2G). The kirigami structural design not only conferred high stretchability to rigid GaN-based μ-LED arrays but also extended it to 3D structure design for arranging hidden pixels, resulting in a display device that could output stable image quality even under high strain.

Moreover, highly complex kirigami patterns can be realized using computer-aided design methods to match the morphology of 3D objects with various curvatures. Lee et al. [43] proposed the concept of computational wrapping to transform a nonstretchable 2D flexible device into a 3D conformal device. A 3D surface was divided into a number of interconnected 2D meshes by computer-aided design, and the distances and bending angles between the 2D meshes were optimized. This design allowed both rigid and brittle materials to completely cover and tightly wrap the nonzero Gaussian curvature surface without apparent fracture.

Currently, flexible/stretchable optoelectronic devices achieved by structural engineering are mainly based on island-bridge structure or kirigami structure with stress–strain relief property combined with serpentine wire interconnections. The LEDs or PDs are placed in pixel islands where the strain is very low, and the serpentine wires used to interconnect the islands are used as strain dissipation zones during stretching. However, structural engineering also suffers from small elastic deformation and strain dissipation failure during stretching and faces the challenges of fragility during stretching and torsion, and excessive loss of device density and resolution [53].

#### Intrinsically stretchable materials design

Although structural and strain engineering are effective strategies for imparting stretchable property to rigid materials, the achieved mechanical properties are low and inconsistent in different directions. In addition, drawbacks such as low device density and complex processes also impede the development of stretchable optoelectronic devices. Compared with the physical or structural stretchability, the design of intrinsic stretchability is a more rational method for developing stretchable optoelectronic devices.

In order to confer intrinsic stretchability to the functional layers of the devices, it is crucial to modulate the intra- and interchain interactions of polymers to provide a pathway for strain dissipation. These interactions not only determine the conformation and dynamic properties of polymers but also affect the stacking mode and aggregation of polymers. There are 2 main approaches to achieve intrinsic stretchability. The first approach involves backbone engineering and side-chain modification through the introduction of flexible units or dynamic bonds (e.g., hydrogen bonds and metal–ligand bonds), etc. [54–60]. The second method need to dope additives, nanofillers, cross-linkers, and other functional agents into elastomers [61–63].

Backbone engineering: The chemical structure of the polymer backbone directly influences the molecular stacking pattern and the corresponding film morphology. It has been shown that decreasing the overall crystallinity of a polymer film increases its crack onset strain, which is attributed to the strain dissipation through amorphous regions [64]. Chain dynamics can be increased by introducing flexible spacer units on the backbone chains. Results have shown that reduced elastic modulus and increased crack onset strain are observed with increasing content of flexible spacer units [54,55]. However, the flexible spacer units disrupt the charge transport network, leading to a decrease in carrier mobility or luminescence property. Therefore, an appropriate content of flexible spacer units needs to be introduced to balance the stretchable and optoelectronic properties. Mun et al. [56] inserted different flexible spacers into diketopyrrolopyrrole (DPP)-based polymers and observed that with increasing spacer chain dynamics, the modulus and crack onset strain showed a decreasing and increasing trend, respectively (Fig. 2I and J). Liu et al. [55] inserted soft alkyl chains between the thermally activated delayed fluorescence units on the polymer backbones to dissipate strain energy. Moreover, they demonstrated that the long-range charge hopping network formed by thermally activated delayed fluorescence units was not affected by the insertion of alkyl chains below a certain length, and the effect of the inserted alkyl chains on the luminescence property is negligible. They found that when alkyl chains with the length of 10 carbons are inserted, the synthesized polymer achieved a stretchability of 125%, with an external quantum efficiency of 10%. The polymer was used as a light-emitting layer to construct intrinsically stretchable OLED, which exhibited a high external quantum efficiency of 3.3%, a current efficiency of 10.2 cd A^−1^, a low turn-on voltage of 4.75 V, and a skin-like stretchability of 60%.

Apart from the introduction of flexible spacer units, dynamic bonds are used as noncovalent cross-linking centers, which serve as strain dissipation pathways through the breaking of these reversible bonds. Kang et al. [65] designed and synthesized an elastomeric film with a multistrength hydrogen bonding network with a high stretchability of 1,200% and a toughness of 1,200 J m^−2^. In this polymer film, weak hydrogen bonds were used to dissipate strain energy during stretching, while strong hydrogen bonds were used to enhance the toughness of the film (Fig. 2K). In addition, the film could be self-healable, due to the reconfiguration of the hydrogen bonding network.

Side-chain modification: In addition to backbone engineering, polymer side chain modification is also one of the strategies to achieve intrinsic stretchability. Side chains not only increase the solubility of conjugated polymers but also influence the conformation of the polymers as well as the molecular stacking pattern in the thin-film state. Wang et al. [58] revealed that the stretchability is highly dependent on side-chain dynamics. At temperatures below the glass transition temperature (*T*_g_) of the side chains, all chains were nearly frozen, and the film exhibited brittleness. The film generated cracks and propagated rapidly when being stretched, thus impeding charge transport. When the temperature was above the *T*_g_ of the side chains but below the *T*_g_ of backbone chains, the side chains were unfrozen, offering stretchability to the film. Meanwhile, the backbone chains remained in the glassy state, which contributed to form tight π-π stacking, thereby ensuring charge transport. When the temperature exceeded the *T*_g_ of backbone chains, the chains freedom of the backbone increased, somewhat disrupting the π-π stacking and thus hindering charge transport.

Currently, there are 3 main strategies for side-chain modification, which are flexible nonconjugated side chain, side chain containing dynamic bonding sites, and biaxially extended conjugated side chain. Zhang et al. [59] found that the *T*_g_ and elastic modulus of the DPP-based polymers decreased with increasing side-chain length (Fig. 2L). However, due to the large steric hindrance caused by long side chains, the rotation of backbone chains was hindered, making them difficult to rotate parallel to the direction of strain. Therefore, the side-chain length needs to be moderate to obtain high stretchability.

Compared to flexible side chains, side chains containing dynamic bonding sites can have additional strain dissipation mechanism through the breakage of dynamic bonds. Moreover, the dynamic bonds on the side chains promote the construction of the dynamic cross-linked network, which facilitates the reconfiguration of the charge transport network under stretching. Gasperini et al. [60] introduced 2,6-pyridinedicarboxamide into the side chains of DPP and proved by strain-dependent Fourier transform infrared spectroscopy that the disruption of hydrogen bonds between polymer side chains was one of the pathways of strain dissipation (Fig. 2M). In addition, the temperature of hydrogen bonding network reconstruction was more favorable to strain energy dissipation as it was closer to room temperature.

In addition, the introduction of large biaxially conjugated side chains on the backbone chains results in efficient side chain interdigitation to form physical cross-linking network, which not only allows for a high tolerance to strain but also provides relay sites for interchain charge hopping, thus facilitating charge transfer under stretching [66].

“Doping” strategy: Apart from optimizing the chemical structure of backbone chains and side chains, the “doping” strategy of adding a second component is also a promising strategy. Recently, the main approaches include the addition of small-molecule additives and blending with elastomers. Kim et al. [61] reported that the addition of small-molecule nonionic surfactants could improve the mechanical properties of conjugated polymers through plasticizing effect. The addition of Triton X, a small-molecule nonionic surfactant, to Super Yellow (SY), a luminescent polymer, Triton X could increase the free volume of SY by reducing polymer interchain interactions, thereby improving the stretchability of SY. The addition of Triton X to the hole transport material poly(3,4-ethylenedioxythiophene):polystyrene sulfonate (PEDOT:PSS) prevented strong electrostatic interactions between PEDOT and PSS, which induced a phase separation of PEDOT:PSS, enabling PEDOT to form a nanofibrous structure. The nanofibrous PEDOT could possess a more continuous hole transport pathway along with improved stretchability. They constructed intrinsically stretchable OLEDs using Triton X-doped light-emitting and hole transport layers, which could emit light at a high strain of 80%. The maximum luminance was 4,400 cd m^−2^ and could withstand up to 200 repeated cycles of stretching.

However, the addition of small-molecule plasticizers can only increase the stretchability of the conjugated polymers without improving their optoelectronic properties. Zhang et al. [62] developed a strategy for blending SY with polyurethane (PU) elastomer. The spontaneous phase separation of SY and PU allowed SY to form an interpenetrating nanofibre structure, which not only dramatically improved the stretchability but also shortened the π-π stacking distance and thus facilitated charge transport. Moreover, blending with wide bandgap polymer can dilute both the transport and trap states. From the statistical relationship between free charge carriers and trap charge carriers, it can be concluded that the simultaneous dilution of both states can lead to a marked enhancement of the trap-limited current. As a result, it is confirmed that blending luminescent polymer with suitable elastomer can simultaneously enhance the stretchability and carrier transport density through nanoconfinement effect and charge-trapping dilution effect. The intrinsically stretchable all-polymer LED (PLED) was constructed, with an unprecedented high brightness of 7,450 cd m^−2^, as well as a high current efficiency of 5.3 cd A^−1^ and a high stretchability of 100%. Similarly, Liu et al. [63] constructed a self-assembled 3D penetrating network based on high-molecular-weight phenylenevinylene (L-SY-PPV) and polyacrylonitrile (PAN) using the nanoconfinement effect, which led to an increase in the stretchability from 20% to 100% and simultaneously achieved a 5- to 6-fold enhancement in the carrier mobility (Fig. 2N to P).

### Interface adhesion design

In biointegrated systems, optoelectronic devices need to establish stable conformal contact with the surface of biological tissues in order to apply precise stimuli or obtain high-fidelity biological signals. Achieving stable conformal contact requires not only excellent stretchability of the devices but also a tight and stable interfacial adhesion with biological tissues. The key to device–tissue interfacial adhesion is to achieve mechanical coupling between the devices and the tissues in wet and dynamic environment. However, the modulus difference between biological tissues and optoelectronic devices is high (e.g., the Young's modulus of human skin and brain is about 140 to 600 and 1 kPa respectively, while the modulus of polyimide (PI), a flexible substrate commonly used for optoelectronic devices, is 8 GPa [1]), so it is challenging to achieve robust adhesion between optoelectronic devices and biological tissues. In addition, device–tissue interfaces also need to possess good light transmission and ionic conductivity to enable optical, electrical, or chemical exchange at the interface. At present, some commercially available bioadhesives suffer from poor adhesion on wet surfaces, low ionic conductivity, and the inability to form conformal contact with complex 3D surfaces [67]. Hydrogel has been widely used as an interface layer between bioelectronic devices and tissues due to its resemblance to the mechanical and chemical properties of biological tissues [68]. According to the adhesion mechanism, the interface adhesion designs can be classified into 3 categories: irreversible covalent adhesion, dynamic covalent adhesion, and noncovalent adhesion.

#### Irreversible covalent adhesion

**Fig. 3.**
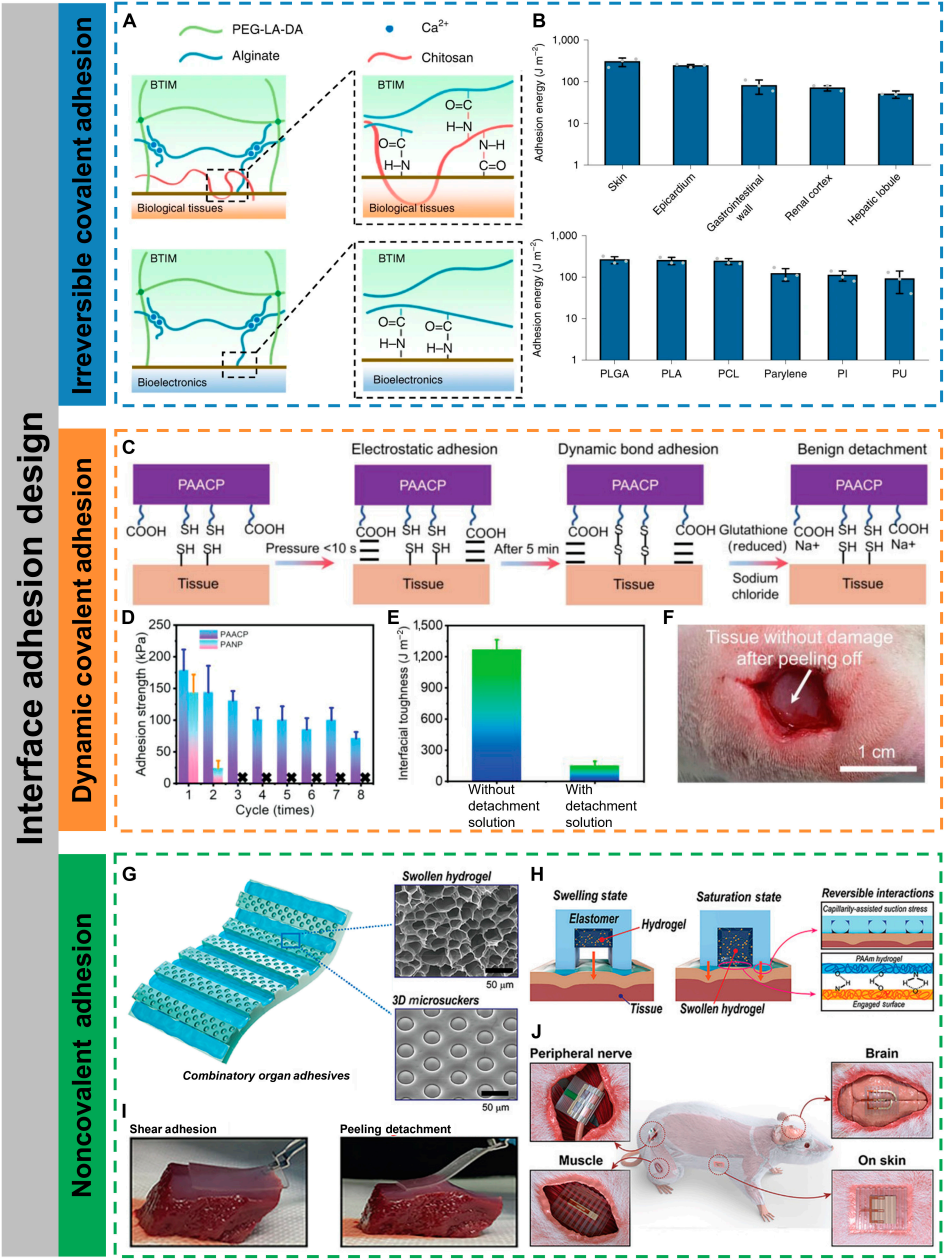
Interface adhesion design. (A) Mechanisms of BTIM bonding to the surfaces of biological tissues (top) and bioelectronic devices (bottom). (B) Adhesion energy between BTIM and a variety of tissues and device surfaces. Reproduced with permission from [67]. Copyright 2021 The Authors, under exclusive license to Springer Nature Limited. (C) Schematic of PAACP adhesion and detachment mechanisms. (D) Adhesion strengths obtained for 8 continuously repeated adhesion tests. (E) Influence of detachment solution on PAACP interfacial toughness. (F) Photograph of muscle without damage after on-demand and benign detachment of PAACP by using detachment solution (implanted into the subcutaneous muscle of the rat for 14 d). Reproduced with permission from [69]. Copyright 2023 Wiley-VCH GmbH. (G) Schematic illustration of the multiscale combinatory organ adhesion with swollen hydrogel and 3D microsuckers. (H) Adhesive mechanism of the combinatory organ adhesion via mechanical interaction of capillarity-assisted suction stress and hydrogel absorption and swelling, which induces multiple-point hydrogen bonds between the hydrogel and proteins on the tissue surface. (I) Demonstration of strong shear adhesion and facile peeling detachment of a combinatory organ adhesion on a liver surface. (J) Schematic illustration of the integrated adhesive electronics adhered to various organs (peripheral nerve, brain, muscle, and skin). Reproduced with permission from [71]. Copyright 2021 Wiley-VCH GmbH.

Irreversible covalent adhesion mainly involves the formation of stable covalent bonds represented by amide bonds at the interface, and the resulting adhesion force is generally several times stronger than the intermolecular interactions. Yang et al. [67] reported a biodegradable and light-curable bioelectronic–tissue interface material (BTIM) composed of polyethylene glycol-lactide acid diacrylate with ionic networks (sodium alginate), which was used as an interfacial layer to achieve strong adhesion between bioelectronic devices and various biological tissues. Figure 3 A shows the mechanism of bidirectional adhesion through BTIM. The tissue surface was precoated with chitosan and coupling agent (1-ethyl-3-(3-dimethylaminopropyl) carbodiimide and sulfated *N*-hydroxysuccinimide). Primary amine groups on the chitosan backbone are covalently bonded to carboxylic acid groups on the tissue surface and on the alginate network to provide strong adhesion. The surfaces of devices were functionalized with amino groups ((3-aminopropyl)triethoxysilane modification) and precoated with the coupling agent. The modified amino group on the device surface is covalently bonded to the carboxyl group on the alginate through the coupling agent. Moreover, alginate can also further enhance adhesion through physical entanglement of chains after cross-linking by Ca^2+^. The adhesion energies of the interface layer to the wet skin surface and the epicardium were 1,300 ± 70 J m^−2^ and 240 ± 20 J m^−2,^ respectively (Fig. 3B). The adhesion energies of the interfacial layer to device substrates such as polylactic acid and PI were 250 ± 50 J m^−2^ and 110 ± 30 J m^−2^, respectively (Fig. 3B).

#### Dynamic covalent adhesion

Dynamic covalent bonds, such as disulfide bonds, are often reversibly broken or reconfigured under certain circumstances. Using dynamic covalent bonds as an interfacial adhesion strategy allows for reversible and repeated adhesion and on-demand peeling without harming biological tissues. Tian et al. [69] reported a nonswelling hydrogel (PAACP) with high cardiac adhesion strength of up to 128 kPa, which was about 10 times higher than that of other reported nonswelling hydrogels, and that could be reusable. The nonswelling hydrogel was made by copolymerizing and cross-linking poly(vinyl butyral) with acrylic acid, gelatin, and chitosan-grafted *N*-acetyl-L-cysteine. To form reversible covalent adhesion with the tissue surface, chitosan-grafted *N*-acetyl-L-cysteine with sulfhydryl groups was used to form dynamic disulfide bonds with the tissue surface (Fig. 3C). The disulfide bonds that can be reconfigured under near-environmental conditions gave the hydrogels regenerable tissue adhesion, and the adhesion strength could maintain above 70 kPa after 8 cycles of repeated adhesion-peeling tests (Fig. 3D). Additionally, separation solutions can be used to disrupt the dynamic disulfide bonding bonds to enable on-demand and noninvasive separation of hydrogels from biological tissues (Fig. 3E and F). Xue et al. [70] designed a hydrogel interfacial layer based on the dynamic interaction of boronate-diol complexation with an interfacial toughness of over 400 J m^−2^. In addition, this hydrogel adhesive layer could also be triggered to separate by the addition of glucose without causing any damage to biological tissues.

#### Noncovalent adhesion

Noncovalent adhesion is based on electrostatic interactions, e.g., hydrogen bonding and π-π stacking. The advantages over covalent adhesion are that no additional coupling agents and complex surface pretreatments are required and rapid adhesion at the interface can be achieved. Although individual hydrogen bonding is weak, the formation of hydrogen bond arrays can compensate for the lack of interaction forces. Kim et al. [71] developed an intrinsically stretchable adhesive layer with synergistic adhesion by electrostatic (hydrogen bonding) and mechanical (capillary-assisted suction) interactions. Inspired by the hierarchical microchannel network of mucus in the toe pads of tree frogs and octopus suction cups, the adhesive layer was embedded with nanoporous hydrogels in microchannels, and the channel walls were constructed with 3D suction cup structures (Fig. 3G). Through the coupling of biofluids on the wet surface of biological tissues, the hydrogels swelled and contacted with the tissue surface to form hydrogen bonding arrays, while squeezing the channel walls to increase capillary suction (Fig. 3H). The adhesive layer could be reversibly adhered and peeled off from the tissue surface and was capable of application to peripheral nerve, brain, muscle, and skin surfaces (Fig. 3I and J).

### Encapsulation design

Implantable optoelectronic devices are in direct contact with organs and tissues in the human body, which renders them susceptible to biofluid erosion. Thus, an encapsulation layer with excellent biofluid barrier property is required to protect the electronic components inside. In addition, the encapsulation layer must be biocompatible to avoid unnecessary toxic damage to tissues. Based on the duration of the implantable optoelectronic devices executing their function in the body, the encapsulation design strategy can be categorized into long-term encapsulation and biodegradable encapsulation.

#### Long-term encapsulation

**Fig. 4.**
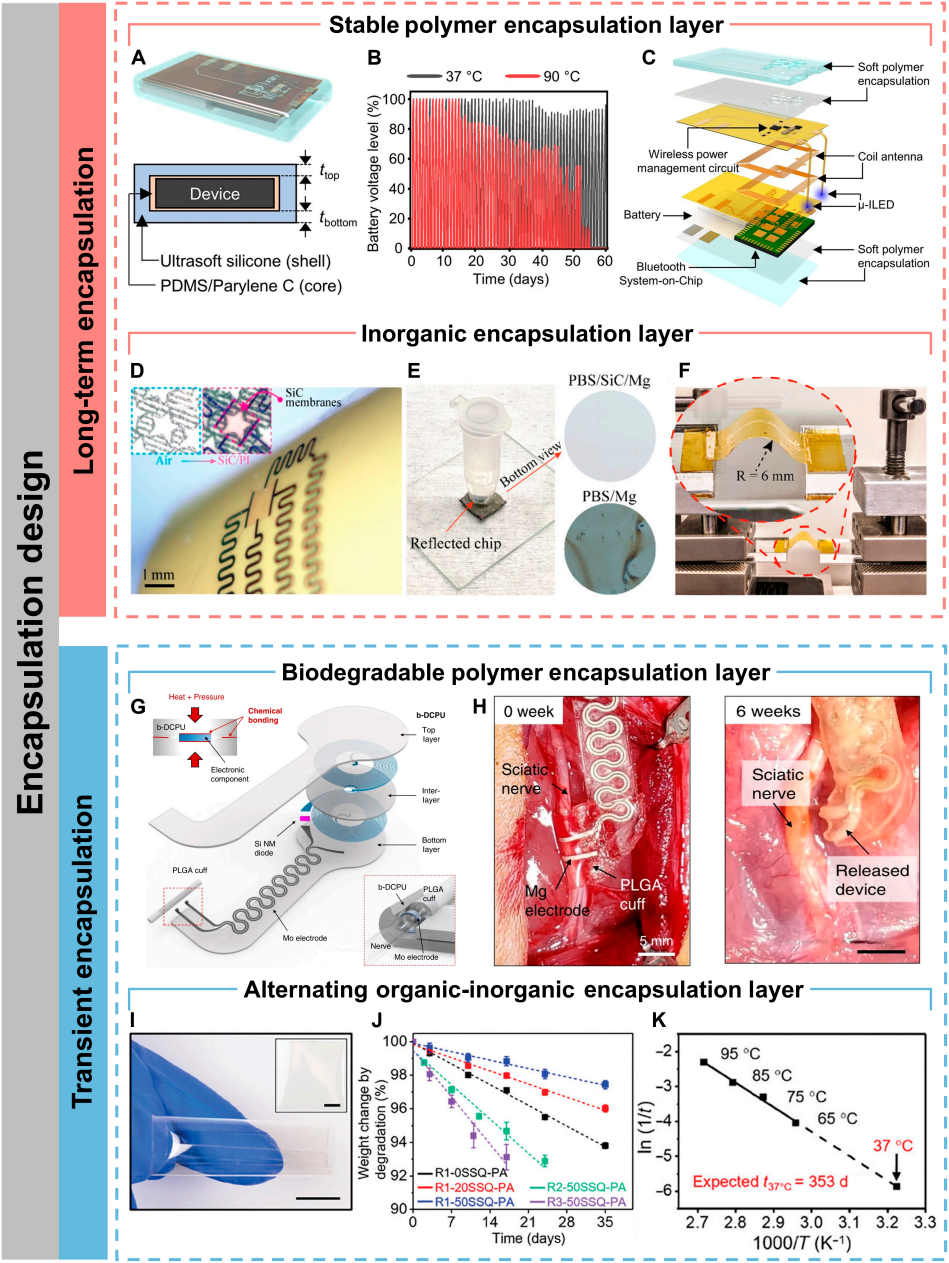
Encapsulation design. (A) Schematic diagram of an implant encapsulated with soft biocompatible polymers (top) and its cross-sectional view (bottom). (B) Battery voltage level as a function of time during repeated wireless charging (60 min) and μ-ILED operating (20 Hz, 10-ms pulse width) after immersing the devices in saline water with temperatures of 37 and 90 °C. (C) Exploded-view schematic diagram of a soft wireless optoelectronic system with bilateral probes, consisting of μ-ILEDs, a power management circuit, radiofrequency coil antennas, a battery, and a Bluetooth Low Energy System-on-Chip. Reproduced under the terms of the Creative Commons CC BY license from [74]. Copyright 2021 The Authors. (D) Flexible SiC-on-PI devices wrapped around a curved surface (diameter = 12 mm). (E) Photograph of SiC/PI immersing at PBS at pH 7.4 and 96 °C. (F) Photograph of SiC/PI upon several bending cycles. Reproduced with permission from [76]. Copyright 2019 American Chemical Society. (G) Schematic illustration of the device consisting of an interlayer (b-DCPU, 50 μm thick), a stimulation cuff (PLGA, 30 μm thick), etc. All parts of the system, excluding the stimulation cuff, are sandwiched between 2 layers of bioresorbable elastomers (b-DCPU, 100 μm). The schematic illustration in the inset shows the contact between the nerve and the stimulation cuff (bottom, right) (H) Images of the release device from the bioresorbable stimulator on the sciatic nerve after 6 weeks. Reproduced under the terms of the Creative Commons CC BY license from [79]. Copyright 2020 The Authors. (I) Photographs of a 3-layer SiON-PA film. Scale bars, 1 cm. (J) Degradation behavior of PA (thickness, 100 μm) with different compositions. (K) Arrhenius plot of the temperature-dependent degradation of 3-layer SiON-PA films. Reproduced under the terms of the Creative Commons CC BY license from [80]. Copyright 2024 The Authors.

Long-term implantable optoelectronics can be used for continuous monitoring of the corresponding pathological data of patients or stimulating tissues and organs for intelligent diagnostic and therapeutic integrated medicine. Long-term implants require the encapsulation layers have excellent long-term biofluidic barrier property, which can effectively block water infiltration as well as ionic diffusion [72]. Soft polymers are ideal for the encapsulation of implantable optoelectronic devices due to their excellent flexibility and even stretchability, which allows for optimal contact with biological tissues. Kim et al. [73] used a Parylene C/SU8-2 bilayer encapsulation layer to minimize the effects of biofluids such as external moisture and sweat due to its excellent water resistance. The encapsulation layer could retain its water barrier capacity for more than 118 h at 40 °C. The encapsulation layer also provided excellent strain isolation ability, allowing for preventing mechanical damage and failure of quantum dot LED display arrays in harsh mechanical environments such as extreme rolling, bending, and wrinkling. Kim et al. [74] used a bilayer polymer encapsulation strategy (Fig. 4A), where the inner encapsulation layer consisted of poly(dimethylsiloxane) (PDMS) and Parylene C, which acted as a biofluid barrier. The outer encapsulation layer was made of Ecoflex GEL, an ultrasoft polymer for mechanical buffer and seamless integration into the tissues. The encapsulated devices remained stable for 55 d after immersion in phosphate-buffered saline (PBS) at 90 °C (Fig. 4B), and a lifetime of approximately 1 year at 37 °C was obtained based on Arrhenius extrapolation. Wireless-powered μ-LEDs were encapsulated with the bilayer polymer and integrated to the skulls of freely moving rats for optogenetic stimulation (Fig. 4C). In addition, optoelectronic devices inevitably suffer from mechanical damage after long-term operation in a stretched environment, leading to performance degradation or even failure. Self-healing materials are expected to achieve morphological and even functional reconstruction after mechanical damage by mimicking the recovery properties of human skin after injury. Son et al. [75] developed a PDMS–MPU_0.4_–IU_0.6_ self-healing encapsulation layer embedded with a conductive network of carbon nanotubes or silver nanowires (Ag NWs), which could reconfigure to restore its encapsulation and conductivity after mechanical damage. The Ag NWs/PDMS–MPU_0.4_–IU_0.6_ layer conformally adhered to the skin due to its low modulus and continuously performed LED actuation, real-time strain, and electrocardiogram monitoring.

Compared to polymers, inorganic materials have superior biofluid barrier property, but the rigidity of inorganic materials limits the application of inorganic encapsulation layers to highly dynamic organs. Phan et al. [76] selected single-crystal silicon carbide (SiC) with very low water permeability, ionic diffusivity, and hydrolysis rate and developed a technique for physically transferring SiC nanofilm (NM) as an encapsulation layer on a PI flexible substrate (Fig. 4D). The SiC NMs were exposed to PBS at 96 °C with an etching rate of 0 nm/d, and no water permeability was observed for at least 60 d (Fig. 4E). In particular, when the SiC NM transferred onto the flexible PI substrate was buckled with a bending radius of 6 mm, no obvious cracks were observed (Fig. 4F). Inorganic encapsulation materials can also be flexible by an ultrathinning process. Chiang et al. [77] prepared a robust, ultrathin (1 μm), and bendable silicon dioxide (t-SiO_2_) layer by thermal growth. The encapsulation layer exhibited no pinhole defects and had an expected lifetime of up to 60 years. In addition, unlike polymer-based encapsulation, t-SiO_2_ layer was also reliable when using a direct current (dc) bias voltage to power the devices. Finite element analysis showed that the maximum strain in the t-SiO_2_ layer is less than 0.2% at a bending radius of 2.5 mm, which was below the fracture limit (~1%).

#### Transient encapsulation

Some implantable optoelectronic devices for clinical use do not need to operate for a long period, so it is crucial to develop optoelectronic devices that can spontaneously degrade in the biological environment after the operational period in order to avoid the risk of syndromes associated with surgical procedures. This requires encapsulation layers that possess excellent biofluidic barrier property during the operational period of the devices and are biodegradable after the operational period. Choi et al. [78] exhibited a fully implantable and bioresorbable cardiac pacemaker using a widely used polyester-based bioresorbable polymer, poly(lactic-co-glycolic acid) (PLGA), as an encapsulation layer. PLGA can dissolve in vivo by hydrolysis to its monomers, glycolic and lactic acid, which are harmless and metabolized by human body. After immersion in PBS solution at 37 °C, most of the constituent materials were dissolved within 5 weeks, and the remaining residues completely disappeared after 7 weeks. Choi et al. [79] also synthesized a biodegradable dynamic covalent PU (b-DCPU) as an encapsulation layer (Fig. 4G), and the implanted devices encapsulated by this layer operated normally in mice for more than 30 d, showing some degradation at around 6 weeks (Fig. 4H). Hu et al. [80] developed an alternating multilayer organic–inorganic film composed of polyanhydride (PA) and silicon oxynitride (SiON) to serve as a biofluidic barrier encapsulation layer that could both avoid premature degradation of electronic components and allow spontaneous biodegradation after its expected lifetime (Fig. 4I). The degradation rate can be easily regulated by varying the amount of cross-linkers (Fig. 4J). The excellent biofluid barrier property of the encapsulation layer was attributed to the misalignment between the discontinuous defects in the SiON layer, which resulted in a very tortuous path for water infiltration. Moreover, the encapsulation layer could degrade completely at 37 °C over a period of about 353 d (Fig. 4K). Generally, the biodegradation lifetime of alternating organic–inorganic encapsulation layers is slightly longer than that of pure polymer encapsulation layers, so the encapsulation strategy can be chosen to match the biodegradation time scale as closely as possible to the time scale over which the devices need to perform functions.

## Applications of Biointegrated Optoelectronics for Cardiac Healthcare

CVDs have one of the highest mortality rates in the world, and the incidence is increasing every year, but 90% of CVDs can be curable at an early stage [16,81,82]. Biointegrated flexible or stretchable optoelectronic devices play an important role in novel patient-friendly cardiac monitoring and therapy. In terms of cardiac physiological signal monitoring, flexible or stretchable optoelectronic devices offer the opportunity for imperceptible, real-time, and continuous monitoring physiological parameters, which provide accurate guidance for clinical diagnosis and practice. For the cardiac disease treatment, the development of optogenetics with very high spatial and temporal resolution and cellular selectivity has injected new vigor [83]. Implantable optoelectronic devices can overcome the shortcoming of insufficient light penetration ability and have a wide range of applications in optogenetics-based cardiac therapy. In addition, nongenetic cardiac stimulation is developing in parallel to achieve precise light-controlled electrical stimulation without the need for complex genetic engineering.

### Biointegrated optoelectronics for monitoring cardiac physiology

Physiological parameters such as SpO_2_, BP, and HR are commonly used to clinically assess the vital signs and cardiovascular functions of patients [84]. Although mature detection approaches have been developed, such as cuff-based BP meters, electrocardiographs, and nuclear magnetic resonance imaging technology, they are intermittent, bulky, and activity-restricted to measure and collect signals. In order to continuously monitor these physiological signals without disrupting daily life, wearable or even implantable sensing devices are required. Among them, PPG is based on optoelectronic technology to obtain SpO_2_, BP, and HR data. The PPG sensor consists of at least 1 LED and 1 PD, where the LED acts as a light source and the PD detects the transmitted or reflected light. Since the diastolic and systolic activity of the heart causes changes in the blood volume in the vascular tissue, the intensity of the transmitted or reflected light detected by the PD varies with the change of blood volume [85].

#### Blood oxygen saturation

A typical PPG sensor used for SpO_2_ monitoring is pulse oximeter. It is based on the different light absorption properties of oxygenated (HbO_2_) and deoxygenated (Hb) hemoglobin in the blood at specific wavelengths and assesses the oxygen saturation according to the Lambert–Beer's law by making optical measurements at 2 different wavelengths [86]. In SpO_2_ measurements, incident light is absorbed and decayed by pulsatile arterial blood, nonpulsatile venous blood, capillary blood, etc. [86]. Then, the light reaching the PD after decay is amplified and converted into an electrical signal, which consists of a rapidly varying alternating current (ac) component due to the changing absorption of light by Hb and HbO_2_ containing in pulsatile arterial blood, and a steady-state dc component from nonpulsating components [87]. Accurate SpO_2_ measurements require large signal-to-noise ratio. Therefore, large ac and low dc signal amplitudes are highly needed. The ac and dc signal amplitudes recorded by the pulse oximeter under a given condition may differentiate at distinct sensing positions, which is mainly attributed to the different thicknesses of the epidermal tissues surrounding the blood vessels at different sensing locations [88]. Khan et al. [89] measured the ac and dc signal amplitudes in reflective mode at 8 different locations in the human body. Maximum ac and dc electrical signal amplitudes were recorded on the forehead, with a maximum ac signal current of 20 nA for red light and 60 nA for near-infrared light. In addition, the strain at the forehead region is low, and therefore, the motion artifact is relatively small. These make the forehead the best sensing location for oximetry. The lowest ac signal intensity was found at the wrist, where the value was almost half of that recorded on the forehead. In addition, the type of light source can also affect the accuracy of the results. Wavelengths should be chosen where the molar extinction coefficients of Hb and HbO_2_ possess adequate contrast. Based on this, the red light (620 to 690 nm, *ε_Hb_*/*ε*_*HbO*_2__ > 6) combined with green light (470 to 550 nm, *ε_Hb_*/*ε*_*HbO*_2__ < 2) or near-infrared light (740 to 950 nm, *ε_Hb_*/*ε*_*HbO*_2__ < 3) is usually chosen [90].

**Fig. 5.**
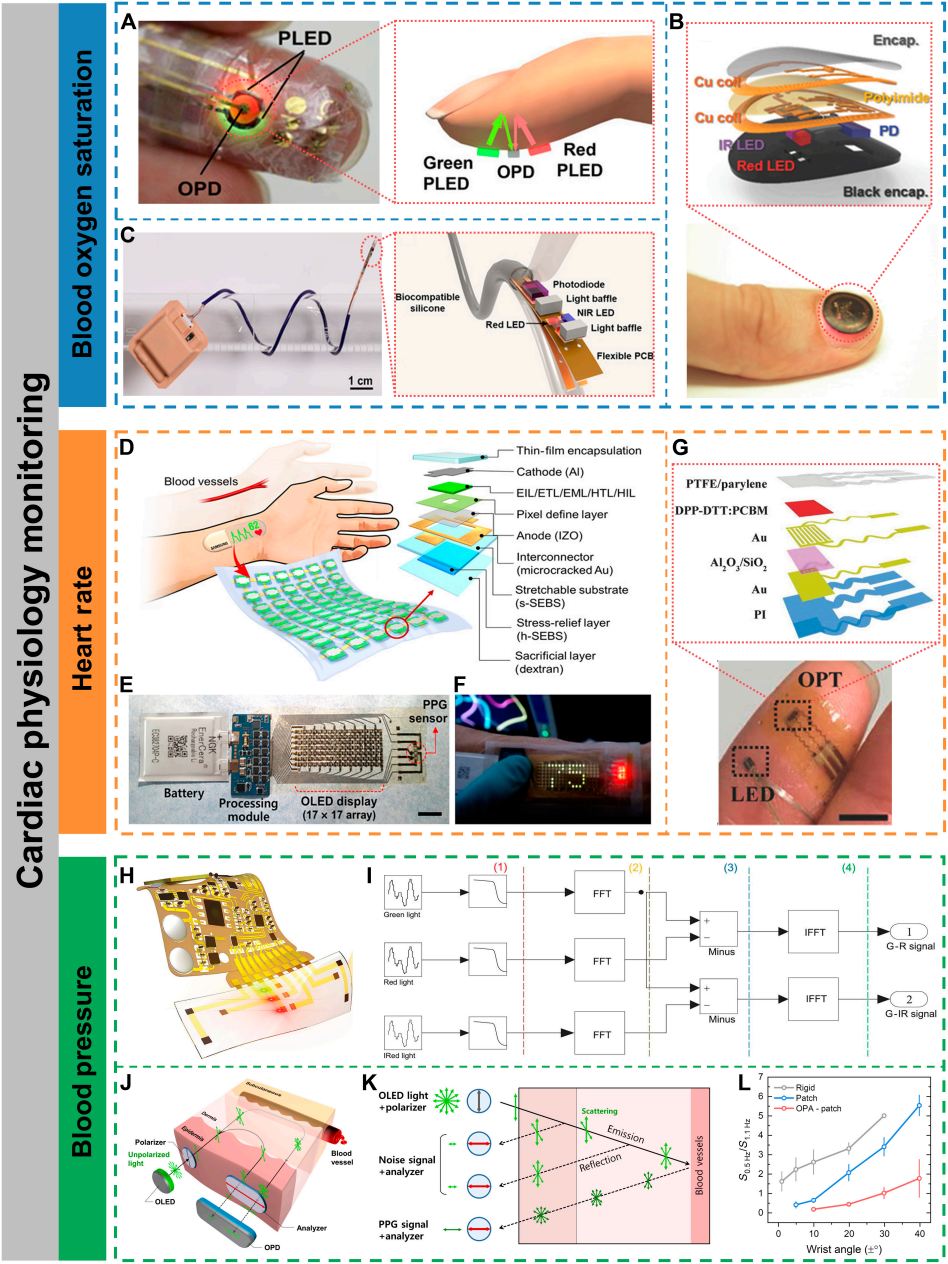
Cardiac physiology monitoring. (A) Photograph of a finger with the ultraflexible organic optical sensor attached (left) and operation principle of the reflective pulse oximeter (right). Reproduced under the terms of the Creative Commons CC-BY-NC license from [92]. Copyright 2016 The Authors. (B) Image of a device during operation while mounted on a thumbnail (bottom) and exploded-view schematic illustration of the various constituent layers of a millimeter-scale, NFC-enabled pulse oximeter device (top). Reproduced with permission from [94]. Copyright 2016 WILEY-VCH Verlag GmbH & Co. KGaA, Weinheim. (C) Image of a catheter oximeter wrapped around a glass rod (left) and the enlarged image of the sensor probe encapsulated with transparent, biocompatible silicone (right). Reproduced under the terms of the Creative Commons CC-BY-NC license from [95]. Copyright 2021 The Authors. (D) Schematic of an SHP attached to the forearm to measure the HR from the wrist (left) and schematic layout of a single pixel on the stretchable substrate (right). (E) Digital image of a fully integrated SHP comprising an organic stretchable PPG sensor, stretchable OLED display, processing module, and thin bendable battery (scale bar, 1 cm). (F) Digital image of the SHP under operation. The HR measured by the PPG sensor is displayed in real time on the green OLED array. Reproduced under the terms of the Creative Commons CC-BY-NC license from [96]. Copyright 2021 The Authors. (G) Photograph of a finger covered with the epidermal PPG sensor (scale bar, 5 mm) (bottom) and schematic of the device structure of the flexible OPT (top). Reproduced with permission from [97]. Copyright 2017 WILEY-VCH Verlag GmbH & Co. KGaA, Weinheim. (H) Schematic illustration of the skin-like device integrated with a flexible circuit for monitoring arterial pressure. (I) Processing steps of the optical difference in the time domain (FFT, fast Fourier transform; IFFT, inverse fast Fourier transform). Reproduced under the terms of the Creative Commons CC BY license from [101]. Copyright 2020 The Authors. (J) Schematic of a polarization-selective sensor. (K) Reflected light in a sensor with an orthogonal polarizer–analyzer pair. (L) Ratio between the peak intensity at ~0.5 Hz (motion artifact) and ~1.1 Hz (heartbeat) during wrist movement over wrist angles ranging from −1° to +1° (±1°), −5° to + 5° (±5°), −10° to +10° (±10°), −20° to +20° (±20°), −30° to +30° (±30°), and −40° to +40° (±40°). Reproduced under the terms of the Creative Commons CC-BY-NC license from [102]. Copyright 2022 The Authors.

Lochner et al. [91] first integrated all-organic red and green LEDs and PDs to construct a transmissive pulse oximeter. The high irradiance of the OLEDs compensated for the lack of transmitted light due to blood absorption. The prepared all-organic optoelectronic oximetry sensor was compared with a commercially available pulse oximeter, and the SpO_2_ error was only 2%. However, the OLED was inflexible and unconformable with human body. Later, Yokota et al. [92] integrated red and green polymer LEDs (PLEDs) and organic photodiodes (OPDs) to develop an ultrathin and stretchable optoelectronic skin for reflective pulse oximetry (Fig. 5A). The optoelectronic system was only 3 mm thick and could withstand multiple strains of up to 60%. The encapsulation layer consisted of alternating organic and inorganic films (500-nm-thick Parylene and 200-nm-thick SiON), which ensured the device operated properly in atmospheric conditions for several days. The device could be laminated directly to organs and senselessly monitor and display SpO_2_ levels during and after surgery.

Optoelectronic devices based on inorganic materials, such as III-V group or silicon-based materials, have superior optoelectronic properties but are inherently brittle and cannot be well integrated with the human body. Li et al. [93] achieved strain isolation through a smart all-in-one suspension structural design, which endowed the inorganic optoelectronic devices with the ability to be fully conformable with the skin and reduce motion artifacts. Specifically, GaAs-based red and infrared LEDs and silicon-based PDs were heterogeneously integrated on the same substrate by stamp-assisted transfer printing and liquid transfer printing methods. The substrate thickness was also reduced via nanodiamond thinning technique to reduce the device thickness to 20 μm. Finally, it was placed in a viscous fluid (liquid PDMS) environment to prepare a free-suspension island for self-adaptive strain isolation and encapsulated with soft materials to form a soft chamber. The all-in-one suspension structure also reduced noise artifacts and signal instability caused by optical path interference due to skin deformation. The prepared device changed only 0.1% SpO_2_ in the stretched state.

The miniaturization of devices allows for a reduced requirement for stretchability. Kim et al. [94] focused on miniaturization and high integration of pulse oximeter to provide more comfortable and senseless long-term monitoring. The key to the miniaturized design was the use of a millimeter-scale bilayer loop antenna instead of a nonstationary multivibrator circuit on the basis of a near-field communication (NFC) system to reduce inductance and crosstalk between circuits. A double-layer interconnect matrix on a thin substrate provided compact electrical wiring between electronic components. PDs that located between 2 red and near-infrared LEDs captured the backscattered light with a spacing distance of 2 mm to allow for adequate data quality with minimal spacing. Additionally, the low modulus (1.17 kPa) and strong adhesion of the encapsulation layer provided stable and robust conformal contact, thereby virtually eliminating motion artifacts. The miniaturized pulse oximeter had an overall size smaller than a US penny and could be integrated onto a fingernail for wireless and battery-free SpO_2_ monitoring with a measurement standard deviation under 1.5 (Fig. 5B).

Apart from patch-type wearable devices, pulse oximeters can also be implantable to achieve higher precision monitoring of intravascular oxygen levels. Lu et al. [95] reported a flexible catheter-type oximeters for wireless, real-time, and consecutive measurement of SpO_2_ with clinical-grade precision. The constructed oximeter consisted of 3 parts: (a) an optoelectronic probe comprising 2 miniaturized LEDs and silicon-based PDs encapsulated in biocompatible, flexible, and transparent silicone elastomer (Fig. 5C); (b) a Bluetooth module powered by a bendable lithium battery; and (c) a human–computer interaction interface deployed on smartphones or intensive care unit monitors for real-time visualization, storage, and analysis of the data as well as setting the illumination parameters of the LEDs. The catheter-type oximeter could be implanted and sutured to the rat heart surface, and the SpO_2_ data matched those tested by a clinical blood gas analyzer.

#### Heart rate

The PPG sensor can monitor HR along with SpO_2_. Each heartbeat is accompanied by the pumping of oxygenated blood into the blood vessels and the return of deoxygenated blood. Therefore, HR can be obtained from the change in HbO_2_ and Hb with each heartbeat. Lee et al. [96] developed a standalone health patch (SHP) for real-time monitoring and display of HR by integrating a stretchable OLED array and a PPG sensor on an all-elastomer substrate (Fig. 5D and E). The SHP design was optimized by analyzing the strain distribution on the skin of the forearm through 3D digital image correlation. Guided by mechanical simulations and 2D digital image correlation analysis, a stress-relief layer and stretchable microcracked Au interconnects were introduced to reduce stress accumulation on the pixel array, allowing the device to operate stably at 30% strain. The PPG sensor monitored pulse waves with high signal-to-noise ratios of >21 dB, which enabled obtaining HR data in real time and visualizing through the OLED array (Fig. 5F).

Low-power consumption is also an important goal for wearable or implantable devices. Xu et al. [97] developed a low-power and high-sensitivity organic phototransistor (OPT) to replace conventional PDs for continuous monitoring HR variability (Fig. 5G). By utilizing an organic bulk heterojunction active layer and a dual-layer gate dielectric design, the constructed OPT possessed a low operating voltage of <3 V, a near-infrared light responsivity of up to 3.5 × 10^5^ A W^−1^, and a noise equivalent power of 1.2 × 10^−15^ W Hz^−1/2^, distinctly superior to those of commercially available silicon-based PDs. Moreover, the ultrathin encapsulation allowed the device to be laminated directly to the skin for more reliable and lower-powered continuous monitoring of HR variability compared to commercially available PPG sensors.

Beyond HR monitoring, it is also possible to integrate PPG sensors with NFC, for example, to fabricate optoelectronic systems that can be powered and transmit data wirelessly. Kim et al. [98] also combined color-responsive materials to extend the functionality in sensing skin color and key environmental parameters such as ultraviolet dosimetry.

#### Blood pressure

Unlike PPG-based HR monitoring, which requires only a single PPG signal, BP monitoring requires the integration of PPG sensors with other bioelectrical or electromechanical sensors to extract BP-related parameters, such as pulse transit time and pulse arrival time [99]. Jinno et al. [100] integrated PLED, OPD, and organic photovoltaic to construct a self-powered ultraflexible optoelectronic skin for continuous BP monitoring. However, the signal quality was degraded by the interference of large motion artifacts from skin deformation.

In order to suppress motion artifacts, Li et al. [101] constructed a skin-like ultraflexible optoelectronic system with 3-color dual-channel (Fig. 5H) and introduced the frequency domain optical aberration method, which adjusted the intensity of the incident light to set the dc of the raw PPG signal at the same level, so that the ac of the PPG caused by different colors of light was different (Fig. 5I). As a result, the difference in PPG intensity between green and red/infrared retained the useful AC signal, which could effectively suppress motion-induced noise. The results of BP monitoring with the skin-like optoelectronic system were compared with those obtained by invasive (intra-arterial) BP monitoring in 44 subjects in the intensive care unit, with maximum absolute errors of ±7/±10 mm Hg (static) and ±10/± 14 mm Hg (walking) for diastolic and systolic BP, respectively, for a total monitoring time of >1,500 min. Lee et al. [102] integrated 2 orthogonal polarizers on a skin-conformable PPG sensor to reduce the amount of epidermal scattered light, which is the primary cause of motion artifacts (Fig. 5J and K). Compared to a rigid PPG sensor, motion artifacts were reduced by over 10-fold for the stretchable PPG sensor integrated with orthogonal polarizers on the wrist and allowed for an increase in the angle of wrist motion from less than 1° to approximately 30° (Fig. 5L).

### Biointegrated optoelectronics for cardiac Optogenetics

“Optogenetics”, the technique of controlling the activity of excitable tissues by expressing light-sensitive opsins in target cells, was first proposed by Deisseroth et al. in 2006 [103]. The expressed light-activated opsins serve as ion channels, ion pumps, or signal receptors and can control the activity of cells, tissues, and even whole organisms with high spatial and temporal resolution [17]. Cardiac optogenetics was enlightened in 2010 and has been widely used to regulate cardiac excitability [29]. By delivering photosensitive opsins to cardiomyocytes or nerves associated with cardiac function, it is feasible to modulate the electrical activity of cardiac tissue by light, which can achieve light-controlled HR regulation and cardiac neuromodulation [104–106]. Optogenetics also provides a new interface for noncontact cardiac pacing [107,108], painless defibrillation [109,110], and precise modulation of action potentials [111,112].

#### Regulation of cardiac arrhythmias

Cardiac pacing, which mainly consists of conventional right ventricular pacing and cardiac resynchronization therapy (biventricular pacing), is a common method of terminating arrhythmias. The principle of optogenetics-based cardiac pacing is to deliver a kind of gene encoding a photosensitive ion channel to cardiomyocytes via viral vectors and use light signals to target and control the cardiomyocytes [104]. When irradiated with different wavelengths of light, the cardiomyocyte action potential will change accordingly to trigger myocardial contraction or diastole, thereby inducing the heartbeat to accelerate or decelerate. In contrast to torturous, nonselective, and high-energy-consuming electric stimulation, optogenetics-based cardiac pacing offers the advantages of painlessness, high spatiotemporal resolution, high cellular selectivity, and low energy consumption [113]. Gutruf et al. [20] achieved a lightweight (~110 mg) and implantable cardiac pacemaker without wires and batteries in small-animal models. The pacemaker was designed with a serpentine structure to withstand more than 200,000 multiaxial strain cycles without degrading electrical or optical performance. The pacemaker integrated microelectrodes and μ-LEDs, enabling not only multisite electrical stimulation but also chronic optical pacing on the ventricular myocardium in ChR2-expressing rats. The pacemaker was powered by a magnetic resonant coupling wireless interface, avoiding the extra burden associated with wires and batteries.

**Fig. 6.**
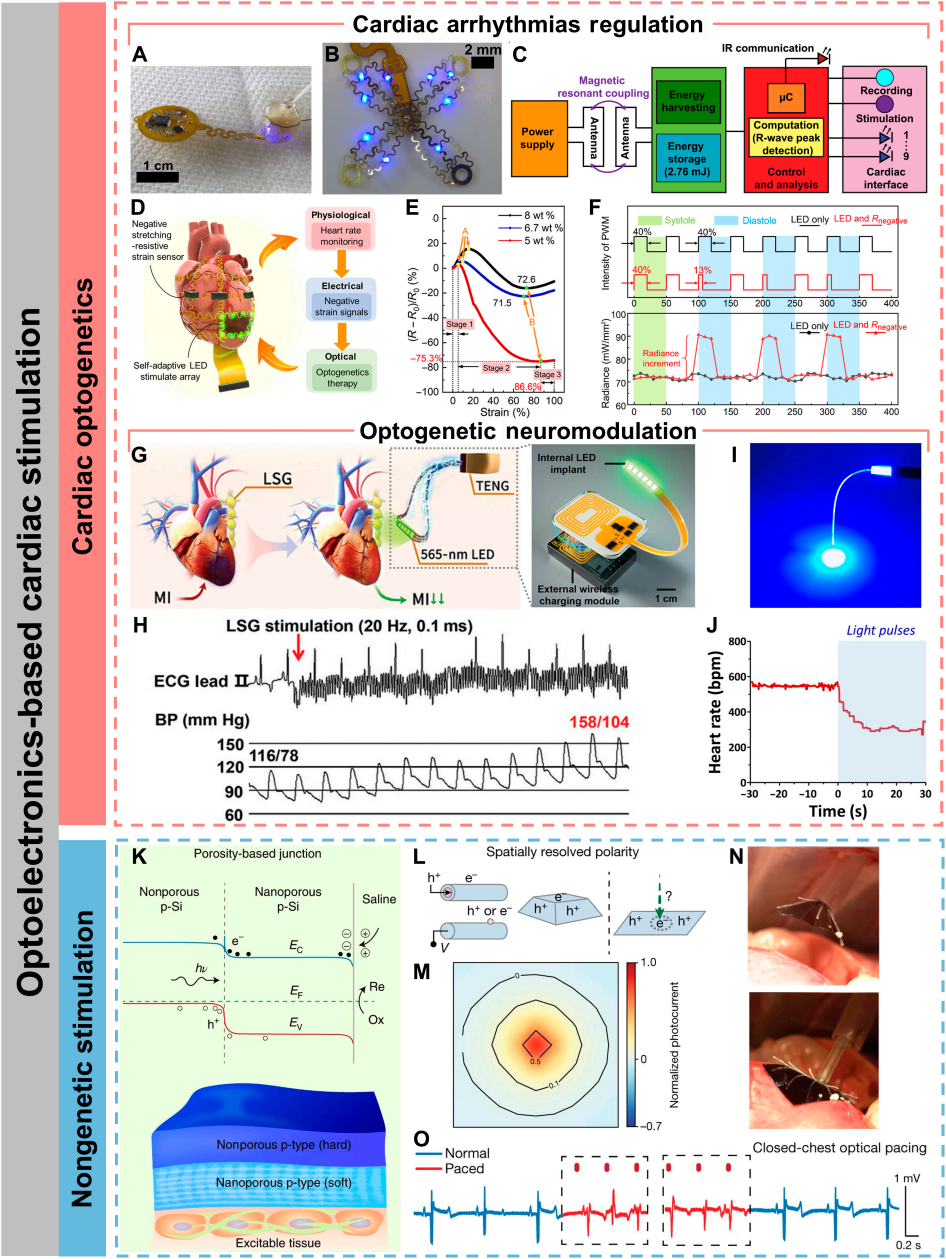
Optoelectronics-based cardiac stimulation. (A) Photographic image of multisite pacing of ex vivo ChR2-expressing mouse heart. (B) Photographic image of μ-ILED and recording array. (C) Schematic diagram of operating electrical principles. Reproduced under the terms of the Creative Commons CC-BY-NC license from [21]. Copyright 2022 The Authors. (D) Schematic illustration of a self-adaptive implantable cardiac optogenetics system based on an original negative stretching-resistive strain sensor array. (E) Resistance characteristics of CNL membranes with different carbon nanotube ratios. (F) The duty cycle settings of the pulse width modulation wave of the optogenetics device with single LED and LED in series with negative or positive stretching-resistive sensor, respectively (top) and radiance of the LED without and with negative stretching-resistive strain sensor in different periods of systole and diastole (bottom). Reproduced under the terms of the Creative Commons CC-BY-NC license from [114]. Copyright 2021 The Authors. (G) Schematic illustration of the optogenetic neuromodulation by the self-powered optical system (left) and schematic diagram of the implantable, battery-free wireless optogenetic system. (H) Representative examples of BP elevation in response to LSG stimulation. ECG, electrocardiogram. Reproduced under the terms of the Creative Commons CC BY license from [118]. Copyright 2023 The Authors. (I) A representative image of a POF exhibiting excellent light conductivity. (J) Representative example of the HR variability in anesthetized. Reproduced under the terms of the Creative Commons CC BY license from [121]. Copyright 2021 The Authors. (K) Schematic illustration (bottom) and energy band diagram (top) of the porosity-based junction. Reproduced with permission from [122]. Copyright 2022, The Authors, under exclusive license to Springer Nature Limited. (L) Optically controlled and spatially resolved polarity enables charge balance, allowing cathodic and anodic processes to occur on the same material surface. (M) The normalized photocurrent mapping against the illumination center. (N) Side-view photographs of the endoscopic device-delivery process (scale bars, 1 cm). (O) Paced electrocardiograph waveforms derived from a fully closed-thoracic procedure. Reproduced with permission from [123]. Copyright 2024, The Authors, under exclusive license to Springer Nature Limited.

Although the work of Gutruf et al. achieved accurate optogenetic pacing for small-animal models, it lacked a closed-loop design for data recording, feedback, and autonomous regulation. Ausra et al. [21] developed a closed-loop cardiac optogenetic pacing system with real-time stimulation, sensing, and computation that could be operated on-demand in free small-animal models. An ultrathin optoelectronic array was fabricated by machine learning-guided serpentine mechanical design and low-cost laser structuring to fit for the topology of the entire heart of a small animal (Fig. 6A). The main body of the device consisted of 4 petal-shaped structures, each containing 1 to 3 μ-LEDs, with 1 petal containing the recording electrode for continuous recording and optogenetic stimulation, resulting in high spatiotemporal accuracy of stimulus delivery (Fig. 6B). In addition, on-board computational capability was used for HR monitoring for immediate feedback and real-time manipulation to ensure millisecond-accurate feedback stimulation. The devices also wirelessly programmed and communicated HR data via infrared uplink. The closed-loop operational design with stimulation, recording, analog-to-digital conversion, and HR calculation processing allows for more precise regulation of HR with optogenetic pacing and defibrillation (Fig. 6C). Hong et al. [114] constructed a cardiac optogenetic system for closed-loop HR monitoring and self-adaptive light-controlled HR modulation through mechanics–electricity–light conversion (Fig. 6D). Different from conventional conductive polymers, which show a rapid increase in resistance due to the disruption of conductive pathways when subjected to stretching, the authors found an interesting phenomenon in which composites based on a high concentration of carbon nanotubes and natural latex (CNLs) show a large decrease in resistance when subjected to stretching. Defined this phenomenon as a “negative stretching-resistive phenomenon”, the CNL film showed a 75.3% decrease in resistance when exposed to 86.6% strain (Fig. 6E). The negative stretching-resistive film was used as a strain sensor and integrated into an implantable closed-loop adaptive optogenetic system, which detected the HR through the resistance change of the negative stretching-resistive material while triggering the self-adaptive optogenetic stimulation module (stretchable LED arrays) at a low-power consumption (<15% duty cycle) in case of an arrhythmia. In the optogenetic module, when the negative stretching-resistive film wrapped on the heart surface was stretched in response to cardiac diastole, the resistance in the circuit was reduced, leading to an increase in the LED light intensity, which enabled self-adaptive regulation of the light intensity and improved the efficiency of optogenetic therapy (Fig. 6F).

#### Optogenetic neuromodulation

Apart from direct photostimulation to cardiomyocytes, cardiac excitability can also be modulated with the help of optogenetics to stimulate nerves related to cardiac functionality. Cardiac autonomic nervous system is associated with cardiac electrophysiology and arrhythmogenesis [115]. Increased sympathetic excitability is recognized as a factor that induces ventricular fibrillation [116]. Previous studies have demonstrated that optogenetic modulation of cardiac sympathetic neurons in the left stellate ganglion (LSG) can accurately and reversibly inhibit cardiac sympathetic hyperactivity, thus increasing ventricular electrophysiological stability and suppressing acute ischemia-induced ventricular arrhythmias [117]. Zhou et al. [118] developed a wirelessly powered flexible LED device that enabled long-term precise cardiac optogenetic neuromodulation for ambulatory canines (Fig. 6G). Long-term optogenetic neuromodulation of the LSG significantly suppressed myocardial-infarction-induced LSG overactivity and remodeling (Fig. 6H). Neuromodulation of the LSG also ameliorated ventricular dysfunction and electrophysiological stability and reduced infarct size as well as ventricular arrhythmias susceptibility.

Parasympathetic nerve (vagal), on the other hand, is a major component bridging the central nervous system and the peripheral cardiac nervous system and also plays a key role in regulating physiological factors such as HR [119]. Vagal excitation can inhibit the pacing activity of the sinus node and thus reduce the HR [120]. Cao et al. [121] prepared a core–shell structured PDMS/hydrogel polymer optical fiber (POF) with low modulus (1.22 MPa) and high stretchability (200%), which allowed for long-term optogenetic stimulation of the vagal in free-moving rodents (Fig. 6I). The POF activated GABAergic interneurons in the left vagal of rodents, which directly resulted in a decrease in HR and exhibit anxiolytic behavior (Fig. 6J).

### Biointegrated optoelectronics for nongenetic cardiac stimulation

Although cardiac optogenetics has very high spatial and temporal resolution, its clinical translation remains challenging. Furthermore, it also involves complex ethical issues due to the requirement for genetic engineering. The recent emergence of nongenetic optoelectronic devices that are capable of converting light into current can efficiently modulate cells and tissues at optical power levels comparable to those applied in optogenetics [122,123]. Nongenetic photostimulation was initially applied primarily to neuromodulation [124]. For example, Ejneby et al. [125] developed an implantable organic electrolytic photocapacitor cuff that converted deep red light into an electrical signal, enabling chronic and conformable photostimulation of the rat sciatic nerve. Recently, nongenetic optoelectronic platforms also hold promise for precise optical stimuli delivery and optical modulation of hearts. Parameswaran et al. [126] used coaxial p-type/intrinsic/n-type silicon nanowires random network and SU-8 polymer support to construct polymer-supported silicon nanowire matrix that could increase the HR of rats during several sessions of low-irradiance laser stimulus (144 mW mm^−2^). The excellent photoelectric conversion ability of the semiconducting silicon nanowires enabled a substantial reduction in the irradiance required for laser stimulus compared to previous work [127,128], thereby reducing the side effects on the human body. Nair et al. [129] used 2D and 3D laser writing to convert a portion of the PDMS substrate into nitrogen-doped cubic SiC (3C-SiC), which was attached to the PDMS substrate via a spongy graphite layer. 3C-SiC exhibited excellent photoelectric conversion activity along with a loose graphite network. Using 3C-SiC/graphite/PDMS as a flexible photoelectrode for pacing an isolated heart, the HR was immediately synchronized with the stimulation rate when a light pulse was applied. Prominski et al. [122] developed a self-limiting stain etching and oxygen plasma treatment process compatible with flexible processes to fabricate nanoporous/nonporous soft–hard heterojunctions in p-type silicon at low cost. Such interesting heterojunctions could form strong photocurrents by reducing the energy level step at the heterojunction interface and the radiative recombination of electrons and holes (Fig. 6K). The minimum laser intensity required for stable rat cardiac pacing reduced to 0.166 mW mm^−2^ attributed to the enhanced photovoltaic ability. Additionally, the heterojunction could respond to near-infrared light with higher penetration capability to generate photoelectric stimuli. Recently, Li et al. [123] investigated porous silicon even further and developed leadless monolithic silicon-based photodiodes as a nongenetic biomodulation platform for multiscale spatiotemporal photostimulation of hearts. The device was only 1 μm thick and weighed less than 0.02 g, which was ^1^/_50_ of the current state-of-the-art pacemakers (≥5 g). The high-resolution and high-precision spatiotemporal photocurrent was benefited from the highly restricted carrier diffusion due to the nanoporous structure (Fig. 6L and M). The device enabled the first optical overlay pacing and multisite pacing of porcine hearts in the closed-chest state using a customized endoscopic operating system (Fig. 6N and O). This leadless and multisite nongenetic photostimulation platform demonstrated the potential to be used for optical pacing and cardiac resynchronization therapy.

Unlike silicon-based semiconductors, Savchenko et al. [130] developed a graphene-based light-controlled actuator that took advantage of graphene's ability to efficiently convert light into current on a femtosecond scale through a hot-carrier multiplication process to achieve an interface for optical stimulation of cardiomyocytes. The efficiency of the applied photostimulation was independent of light wavelength, but it could be used to modulate cardiac activity in zebrafish embryos by varying light intensity. The mechanism of cardiac activity modulation can be attributed to the capacitive effect on the graphene biointerfaces generated by thermoballistic electron clouds. Due to the capacitive coupling between the cell membrane and the graphene surface, a light-induced charge redistribution on the interfaces occurs and cations are displaced, which leads to depolarization of the cardiac cell membrane.

## Summary and Outlook

This review summarizes the advances in wearable and implantable optoelectronic devices for cardiac healthcare. We focused on the stretchable design, device–biological tissues interface design and encapsulation design of biointegrated optoelectronic devices. Then, we moved on to the examples of cardiac physiological monitoring and optogenetic and nongenetic stimulation by biointegrated optoelectronic systems. With this review, we hope to inspire readers on the design strategies for advanced biointegrated optoelectronic devices and ideas for their integrated design as key components of cardiac monitoring and therapeutic systems.

Although remarkable progress has been achieved, there are still many key challenges to be addressed. The majority of current light sources and PDs for cardiac monitoring and therapy are rigid devices based on stretchable structural engineering. However, the major drawback of structural engineering is the low device density, which prevents high-resolution optical stimulus delivery and high-fidelity signal collection. Although intrinsically stretchable optoelectronic devices are theoretically allowed to maintain their optoelectronic performance at high device density, the processes for fabricating high-integration intrinsically stretchable optoelectronic arrays are immature now. In addition, the optoelectronic performance of intrinsically stretchable materials is still not comparable to inorganic rigid materials, especially after repeated deformations. This is because the internal conductive pathways or exciton transport pathways may be irreversibly damaged due to fatigue accumulation, resulting in severe degradation of optoelectronic performance [131]. Therefore, there are still few reports on intrinsically stretchable LEDs and PDs for cardiac monitoring and cardiac optogenetics. Based on these, the development of high-performance intrinsically stretchable optoelectronic materials is of primary importance in the future. Meanwhile, further research on the processing of intrinsically stretchable optoelectronic arrays with high device densities is also an important direction in the future. The key to realizing intrinsically stretchable optoelectronic arrays with high device density is the construction of high-density electrode arrays. This is expected to be achieved by combining photolithography process or high-resolution printing techniques such as electrohydrodynamic printing [132–134]. In addition, it is also possible to introduce dynamic self-healing units to repair the charge transport pathways after undergoing repeated strains [135–137].

It is also challenging to achieve functional interfaces between optoelectronic platform and biological system with uniform, reliable, and time-independent bidirectional adhesion. Interface adhesion that is formed by weak interaction forces such as van der Waals forces can be disrupted due to the potential penetration of cell secretions or inflammatory cells. Covalent bonding provides more robust adhesion but often requires the interface pretreatment as well as the addition of coupling agents. Furthermore, it may cause residue or even tissue damage during peeling. On-demand adhesion based on dynamic covalent interactions or hydrogen bonding arrays may be the focus of the following research [36,138]. Additionally, as for interfacial adhesion layers for optoelectronic devices, it is important to consider not only adhesion energy and interfacial toughness but also to comprehensively optimize light transmission and mechanical properties (strain isolation) to enable efficient light delivery and mechanical match.

In terms of encapsulation design, solely flexible polymer encapsulation materials have a high water-vapor transmission rate and need to be combined with inorganic materials with excellent biofluid barrier property but at the expense of flexibility. Constructing alternating organic–inorganic ultrathin films can ensure excellent biofluid barrier property while maximizing flexibility [80,139]. In addition, most optoelectronic systems used for cardiac monitoring and therapy are temporary implants. Therefore, it is beneficial to develop transient encapsulation technology to avoid the secondary surgical removal. However, the time scale for complete biodegradation is much longer than that associated with stable device operation. The development of stimulus-responsive biodegradable materials may be a rational solution [140].

Furthermore, cardiac physiological monitoring and therapy cannot be achieved by a single optoelectronic device. It also requires integrating energy supply, data transmission, and algorithmic analysis modules to realize an optoelectronic system with the ability to long-term closed-loop operation. Currently, it relies on the serpentine interconnect structure to achieve conformal contact with hearts, but this design is not favorable for further miniaturization of the optoelectronic system and hampers the implantation into the organism in a minimally invasive way. Multifunctional integration techniques for optoelectronic devices, such as monolithic, lateral, and 3D integration on intrinsically stretchable platforms, should be further investigated in the future.

Wireless power supply and data communication are necessary for long-term monitoring and treatment. Promising energy supply solutions are based on miniaturized flexible/stretchable lithium batteries with high power density and long cycle life [141], nanogenerators that can harvest biomechanical energy and convert it into electrical energy, or NFC antennas with dual capability of power supply and data communication [142,143]. However, lithium batteries based on liquid electrolytes have the potential for leakage and short-circuit problems. While solid electrolyte-based lithium batteries are biologically safe, they have low ionic conductivity, resulting in performance deterioration. In addition, nanogenerators are not powerful enough to energize the whole system, and NFC is also limited by transmission distance and sampling frequency [84]. For wearable cardiac healthcare devices, they can also be powered by flexible/stretchable solar cells. Lee et al. [144] have achieved intrinsically stretchable solar cells with power conversion efficiency remaining above 19% at 40% strain, showing great potential for practical applications. However, their poor stability and high cost still restrict wearable applications. Even with robust encapsulation, the morphological stability of the donor–acceptor active layer still remains a tough problem [145]. In addition, besides efficiency and stability, the cost of flexible/stretchable solar cells has received little attention. Synthesis of some highly efficient donor and acceptor materials often requires over 10 steps, inevitably resulting in high costs. In conclusion, flexible/stretchable solar cells as energy supply modules for biointegrated cardiac healthcare devices still need a lot of effort. Therefore, more advanced energy supply methods and data transmission strategies for wearable and implantable cardiac healthcare systems need to be further explored. Multiplexing techniques and edge computing also provide opportunities for real-time transmission and processing of massive amounts of data. However, there are only a few examples of combining optoelectronic systems with big data models and deep learning for assisted diagnosis. In the future, artificial intelligence-based big data models and machine learning are expected to be integrated to help predict and diagnose diseases and provide personalized treatment advices to patients [146].

Thermal management of wearable and implantable devices is often ignored. Wearable and implantable devices can generate heat during operation, which may cause discomfort to users. Thermal management of these devices can provide improved comfort and health benefits to users. However, to the best of our knowledge, there are few examples of integrating wearable and implantable optoelectronic devices for cardiac healthcare with thermal management devices. In the future, wearable and implantable optoelectronic devices are expected to be integrated with thermal management modules such as passive radiative cooling materials, evaporative cooling, or heat sinks to provide a more comfortable and healthy experience for users [147]. The breathability, light weight, and stretchability of thermal management devices also need to be further optimized, and integration with sensing and energy harvesting systems is needed to enable greater intelligence.

It is believed that future in-depth research in materials science, fabrication processes, and integration technologies will address the current challenges and further promote the clinical translation of biointegrated optoelectronic platforms toward the next-generation smart healthcare terminals.

## Data Availability

The original data supporting this review are from previously reported references, which have been cited. The processed data are available from the corresponding author upon reasonable request.
